# Creating an (ethical) epistemic space for the normalization of clinical and “real food” oral immunotherapy for food allergy

**DOI:** 10.1177/13634593221109679

**Published:** 2022-07-08

**Authors:** Stephanie A Nairn

**Affiliations:** Université de Montréal, Canada; CHU Ste-Justine, Canada; McGill University, Canada

**Keywords:** clinical practice guideline, evidence based medicine, food allergy, immunotherapy, multi-criteria approach

## Abstract

Researchers and sociologists have argued the consequences of standardization vis-à-vis clinical practice guidelines are diverse and argue they should be explored empirically. Sociologists have also argued that “best evidence” for the development of clinical practice guidelines is not restricted to randomized controlled trials and that other forms of knowledge should be embedded in and inform CPGs. There is little research concerning how other types of knowledge are mobilized and taken up in CPGs. This article presents the results of an ethnographic investigation in Canada between 2015 and 2020 of the development of a clinical practice guideline for immunotherapy for food allergy. My research shows that immunotherapy has become the source of controversy regarding whether immunotherapy should be offered in the clinic or remain experimental and whether it should be offered using food or commercial products. I argue that the clinical practice guideline for oral immunotherapy reaffirms what has been previously noted by sociologists; guidelines can serve normative purposes and are not merely technical documents. This case study is unique as it demonstrates how guidelines can serve as “community-making devices” to consolidate “epistemic communities” through the explicit and formal mobilization of ethical principles alongside other forms of “traditional” evidence. The mobilization of a multi-criteria approach that included ethical principles was mobilized in part to counter the de-legitimization and peripheralization of clinical and real food oral immunotherapy.

## Introduction

Sociologists and medical professionals alike have argued that “best evidence” for the development of clinical practice guidelines (CPG) is not restricted to randomized controlled trials (RCTs). Rather, “other” forms of knowledge (should) be embedded in and inform CPGs and also be publicly debated (Berg et al., 2001) in the process of CPG development. Methodologies to appraise and weigh different kinds of evidence are unfortunately lacking (Wieringa et al., 2018) and there is little research exploring how different or “other” types of knowledge are mobilized in practice and taken up in CPGs.

In order to investigate these mobilization processes, the present article reports on a qualitative/ethnographic investigation of the development of a CPG for oral immunotherapy (OIT) for food allergy in Canada by a group of clinicians, scientists, and methodologists. The OIT CPG development occurred several years after my own exploration of the dynamics of controversies about immunotherapy from the perspectives of clinician-scientists, physicians, and commercial representatives about whether immunotherapy should be offered in clinical care as a treatment prior to regulatory agency approvals and relatedly whether it should be offered using “real food” or pharmaceutical products.

Until approximately the last 7 years, the gold standard for food allergy management has been an avoidance approach, complemented by the administration of epinephrine in the event of life-threatening anaphylactic reactions. According to its practitioners, immunotherapy embodies a new paradigm to food allergy management, namely an “exposure approach.” The goals of immunotherapy vary, but the hope is that through incremental exposures to various allergens, individuals can acquire a “tolerance,” desensitization, develop a sustained unresponsiveness to the allergen and/or improve their quality of life. In January 2020, Palforzia, the first pharmaceutical product for the treatment of food (peanut) allergy was approved by the US Federal Drug Administration. An instance of oral immunotherapy (OIT), the drug consists of capsules that contain varying amounts of peanut protein. Its approval was the culmination of approximately 6 years of experimentation with adolescents and children in clinical trials across Canada, the U.S. and internationally.

The development of Palforzia by Aimmune Therapeutics has *co-existed* alongside the development of real food OIT in clinical and private practices across Canada and the U.S. A recent study showed that 13.8% of allergists were performing IT in either research protocols and/or clinical practice (Greenhawt and Vickery, 2015). Other sources estimate that “several hundred” allergists offer OIT in the clinic (See: https://www.oit101.org/find-an-oit-allergist/). My investigations confirmed that physicians were offering OIT to their patients using real food as one can find in food stores as a treatment prior to Palforzia’s approval by the FDA.

This situation thus involves the simultaneous development of pharmaceutical products and “real food” IT and is the source of on-going controversy in the food allergy domain between different individuals who take different stances regarding IT. There are those who advocate for a strictly experimental approach to the development of IT (i.e. that it should remain in experimental or commercial trials), there are those who have argued IT should be offered as a treatment to patients and does not require further experimentation to demonstrate it “works,” and those who argue that continued research in both clinical trials and clinical IT is useful and necessary. The latter group does not negate the value of additional research about IT, but claims it is not necessary per se to demonstrate that IT “works” and who also claim commercial products are not *necessary* to perform IT. The individuals in each of the camps have engaged in commercial clinical trials for IT.

Within the context of the aforementioned controversy (Callon et al., 2009), the present article argues that the OIT CPG served an overarching purpose of creating a defensible epistemic space that would normalize and legitimize the practice of clinical oral immunotherapy. The creation of an “epistemic space” encompassed all of the activities that were carried out during the development of the CPG and is akin to the notion of a “community-making device” (Meyer and Molyneux-Hodgson, 2010). Community-making devices help create a sense of a (global) collective of people and practices and help to nurture partial or “fragile” connections between various actors. Community-making devices (e.g. conferences and academic journals) (Meyer, 2010) can catalyze and be instrumental in the development of “epistemic communities” (Haas, 1992). Epistemic communities involve networks of professionals with recognized expertise and competence in a particular domain, who share normative and principled beliefs, which can provide “value-based” rationale for (social) action. Shared “beliefs” in the verity and applicability of certain *forms* of knowledge bonds epistemic community members and networks.

The creation of an epistemic space vis-à-vis the CPG for OIT involved the development of shared sets of normative and principled beliefs, that is, the situated co-production of normative commitments. What makes this particular case study unique, was the emergence of a “good fit” (Casper and Clarke, 1998) between the desires of the spearheaders of the guideline to create a space for OIT that did not solely rely on “traditional” kinds of evidence and the methodologists’ broader critique of “traditional” evidence as the sole criterion on which to make healthcare-related decisions. The CPG developers implemented a multi-criteria approach (MCA) that allowed “other” evidence (i.e. ethical principles and stakeholder perspectives) to be mobilized formally and transparently alongside “traditional” evidence during the deliberative process and in the final published recommendations.

The present article will proceed by demonstrating how a “new” epistemic space vis-à-vis the CPG was established by defining (in a normative sense) *why* and *how* IT should be taken up in the clinic and made available to potential patients. This transformation re-affirms what has been previously noted by sociologists and medical professionals that clinical practice guidelines are not merely technical documents outlining care processes (Berg et al., 2001). This case study will show how ethical principles were mobilized formally, and in most cases explicitly during the deliberations by guideline developers and medical professionals practicing IT for food allergy. This case study thus represents a departure from the empirical literature on clinical practice guidelines thus far wherein ethical principles have been studied and defined as “non-evidence” (e.g. Knaapen, 2013) and/or as potentially “illegitimate” “other considerations.”

## Clinical practice guidelines in the literature

This case study can be situated within the broader literature on the emergence of biomedical “standards” as well as the analysis of valuation practices in knowledge making, in particular Health Technology Assessment (HTA) and Clinical Effectiveness Research (CER) (Hoffman, 2018). Valuation frameworks incorporate evidence hierarchies similar to those enacted in the development of CPGs.

Standardization initiatives, in particular evidence-based medicine (EBM) and CPGs, have been critically analyzed by both sociologists and medical professionals. Sociologists have expressed their concerns about the meaning and consequences of standardization practices and processes for the medical profession (Timmermans and Almeling, 2009), emphasizing the disciplinary and de-humanizing consequences of these practices for healthcare. With regards to EBM, Mykhalovskiy and Weir (2004) have classified these criticisms in two categories. The “political economy” critique of EBM argues that guidelines are tools to advance corporate interests and ideologies, as well as tools to rationalize and destabilize medical authority. The “medical humanism” critique conceives of EBM as reducing patients to “passive recipients” of information and subverting the integrity of *clinical* reasoning, by the same token diminishing the importance and *value* of the doctor/patient relationship. Yet, sociological research has also shown that the consequences and practices of standardization are “ubiquitous, diverse and ambiguous” (Timmermans and Almeling, 2009: 25). The use of qualitative and ethnographic methods has permitted sociologists to document how standards, for example, CPGs and medical consensus conferences (Solomon, 2011) are *made* valuable and mobilized in *practice*. Standards require and elicit, “*ad hoc* tinkering, reappropriation, and explanation” (Timmermans and Almeling, 2009).

Few studies have explored the *purposes* of guideline development (Wieringa et al., 2018) and a few contributions have argued that CPGs are not *just* tools to promote the rationalization of medical practice, as per the political economy critique, but incorporate several purposes including *defining* norms, *summarizing* evidence, *formalizing* current consensus and/or *describing* current practices in a *handbook-type* format (Wieringa et al., 2018). CPGs can take on different forms similar to “essays” outlining the philosophies of groups (Timmermans and Berg, 2003: 123). Moreover, CPGs can be developed to impact professional boundaries and organizational patterns (Castel, 2009). For instance, Timmermans and Berg (2003) showed how the Nursing Interventions Classification (NIC) guidelines were developed to *unite* nurses around a common “task package” and to *stake out claims* to specific areas of technical expertise. They argued that guidelines have several purposes and effects including generating a sense of professional identity, the cultivation of unity, scientific “stock taking” and can become rallying points for research, education, and reimbursement decisions.

The notion that CPGs are narrowly technical and logistical documents to make practices more scientific and homogenous has thus been challenged. Similar to other technologies, as shown by Science and Technology Studies (STS), there are many political and ethical assumptions “embedded” (Berg et al., 2001) in the development of CPGs, and therefore, in collective decisions concerning the various aspects of the CPG process. Moreira’s (2005) ethnographic study of CPG development shows how different “repertoires of evaluation” influence the appraisal of evidence, including: usability, acceptability, adequacy, and robustness. These findings contradict the claim that structural frameworks deterministically influence or reflect guideline development processes. Building on these claims, other sociologists (Knaapen, 2013, 2014; Will, 2009) have shown that “non-evidence,” ethical considerations and pragmatic concerns can be integrated and *override* “standard” evidence in the development of CPGs. Of particular relevance to this article, Knaapen (2013) explored how “other considerations” or “non-evidence” (e.g. literature, opinions, ethical principles and clinical practice standards) mediate and/or are mobilized in clinical practice guideline development. Ethical principles espoused via oral statements were shown to promote or support inaction (no intervention) in some instances among guideline developers.

Medical professionals and sociologists alike have critiqued EBM’s “evidence hierarchy” for undervaluing “other” evidence or forms of knowledge. Despite acknowledgment and research showing that “other” social and ethical considerations are integrated into guidelines (Will, 2009), there is little research exploring *how* patients are mobilized in EBM (Timmermans and Almeling, 2009) or precisely *how* social and ethical considerations are valued, appraised, weighed and then translated into CPG recommendations. There is nonetheless no doubt that patients and patient organizations for example, are no longer simply advocates for diseases, but are now involved in the production of evidence themselves via “evidential work” (Akrich et al., 2012; Rabeharisoa et al., 2013). They work toward identifying areas of “undone science” (Frickel et al., 2010). There is also a nascent domain of scholarship that explores the intersections of social and political movements and the involvement of medical professionals as “activists” engaged in evidential work (Joffe and Weitz, 2003). Pushkar and Tomkow (2021) have theorized that medical professionals can leverage their “symbolic power” to confront and resist broader political forces or shifts alongside and in collaboration with patients.

As previously stated, the following article will further explore previous claims regarding the “other” purposes of clinical practice guidelines, and in particular the mobilization of guidelines to counter de-legitimization and normalize a “new” therapeutic approach to food allergy. I will additionally explore how, in contrast to previous sociological research, ethical principles and stakeholder perspectives were *formally* invoked alongside traditional evidence to produce the CPG and create a defensible epistemic space.

## Methodology

My investigation of the clinical practice guideline was preceded by approximately 4 years of qualitative and ethnographic research about IT for food allergy. Prior to the CPG, I engaged in semi-structured in-depth interviews with 31 individuals with various types of engagement in the field of IT. I engaged in over 100 hours of observations at clinical and scientific conferences, informal conversations and reviews of the clinical trials underway for IT.

My observations of the clinical practice guideline process subsequently occurred over approximately 6 months in mid to late 2019. I was invited by the CPG executive group consisting of *n* = 6 clinician-scientists, methodologists and a bioethicist, to observe the CPG process because of my previous research on the evolution of IT. The methodologists were a methodological “support” team that were consulted via INESSS to develop the CPG and were experts in the multi-criteria approach methodology and a bioethicist.

Initially, I was invited to consult on the process of guideline development due to my expertise as a sociologist and because I was a member of the Canadian AllerGEN research network that brought together researchers and clinicians to share work on allergy and immunology. The members of this network were aware of my ongoing research about IT. Eventually, through interactions with the executive team, it was determined that my role would be confined to a non-participant observer in accordance with the ethical requirements of my broader research project. As with previous experiences during interviews with clinicians and scientists, I had to consistently restate in my interactions with interviewees and participants that I was not inferring judgments about the claims or arguments made in favor of various aspects of IT, but was documenting the claims made by all individuals involved.

The CPG was financially supported by the Canadian Society for Allergy and Clinical Immunology (CSACI) and the executive team received “methodological support” from INESSS, an organization that is called upon by different organizations to assess the clinical advantages and costs of medical technologies and treatments for potential use in health and social services (Battista et al., 2009). These two groups coordinated and led the tasks involved with the CPG development. INESSS team members recommended the team adopt the MCA.

I observed several different meetings leading up to the deliberation proceedings including a private meeting held during the annual CSACI meeting in the U.S. during which methodologists explained the multi-criteria grid (discussed below) and clinicians could ask questions and volunteer topics to be discussed at the deliberations. It was explained that the CPG initiation occurred 2 years prior to the deliberations wherein the lead member of the executive committee nominated two clinician-scientists to spearhead the process. They were responsible for seeking out methodological frameworks for CPG development.

Between this meeting and the deliberation meetings for the CPG, I engaged in many informal conversations with the executive committee members to acquire more insight into both the process through which information was gathered from stakeholders and the issues and concerns that were salient in the “pre-development” phases of the CPG. I subsequently observed the 2-day deliberation meeting, taking verbatim notes of the interactions. There were *n* = 23 members of the “deliberation committee,” composed primarily of clinicians and scientists who had different types of engagement with OIT for food allergy (i.e. allergists), two nutritionists, two patient representatives (a parent of a child with food allergy and an adult male with a food allergy), a pediatrician, and two family physicians.

Each member of the committee signed an informed consent document outlining the purposes, risks and benefits of my project. Due to the confidentiality terms of the meeting, I was not permitted to use direct quotes and I thus describe the content of the interactions in my own words with references to the published clinical practice guideline. After the deliberations, I interviewed methodologists and deliberation committee members (*n* = 3) to acquire information about developments in the field and to further understand the methodological framework.

The audio-recordings of the deliberation and pre-deliberation meetings and interviews were transcribed in full and analyzed using a thematic coding procedure (Braun and Clarke, 2012). Many of the themes I identified in previous interviews and observations were still salient during the deliberation meetings and these were triangulated with the findings from the deliberation and interviews. However, new topics and themes emerged, particularly concerning the deliberation framework and with regards to IT. I checked the themes against various sociological explanatory (theoretical) frameworks and discarded some frameworks when it became apparent they were not useful explanatory devices. For example, in my initial analysis I focused on the aforementioned emerging domain of scholarship that examines the “symbolic power” of clinicians and the mechanisms through which this “power” can be leveraged to impact knowledge-making. After consulting with my research participants about this framework, it became evident that it was not representative of different dimensions of the process, and I thus altered my frameworks to account for and emphasize the “good fit” of the MCA (elaborated in the following section) and the notion of an epistemic space for IT in food allergy.

## The context of controversy in the development of IT for FA and scattered claims

As alluded to briefly in the introduction, over the past 7 years there has been an on-going controversy in North America about whether IT is “ready” for clinical practice and this was a primary theme emerging through analyzes of my interviews between 2015 and 2018 (See: Nairn, 2022). Several academic and commercial investigators performing multi-site clinical trials for IT have argued that oral immunotherapy is not “ready” for clinical practice and should only be performed in clinical trials. In addition to publicly funded experimental IT, two biopharmaceutical companies have been developing marketable products for the treatment of food allergy—DBV Technologies and Aimmune Therapeutics. For our present purpose it is sufficient to note that DBV Technologies is developing a VIASKIN “patch” for epicutaneous IT (EPIT) and Aimmune, as mentioned previously, recently received FDA approval for their Palforzia capsule for peanut allergy.

Despite the claims that immunotherapy is not ready for the clinic, some Canadian and (more commonly) U.S. physicians and clinician-scientists have also offered oral immunotherapy in their offices as a treatment for patients. The clinician-scientists offering oral immunotherapy in their offices do not necessarily negate the values of clinical trials for understanding different dimensions of OIT, but insist they are not required to further demonstrate that OIT “works.” Indeed, some clinician-scientists in Canada and the U.S. participate in both clinical trials for IT and provide OIT as a treatment for patients. These practitioners also argue that oral immunotherapy for food allergy does not necessitate approvals of “drugs” because IT involves the consumption of food or food proteins. Physicians offering OIT have argued adamantly that experimental and commercial products are in no way superior to the use of food.

The various defensive postures espoused by the aforementioned groups constitute a scattered discursive space in which the groups make claims about the values of and the superiority of their practices. I use the term scattered to refer to the fact the claims and arguments leveraged by the groups have re-emerged in different fora and at different times over the past 7 years.

The claims made by the groups have also been underpinned by different moral or ethical imperatives. For example, clinicians offering OIT as a treatment have argued it was wrong to deprive patients of the treatment because they knew it worked (i.e. placebos as part of IT trials were considered unnecessary and even “unfair”). In terms of economic considerations, clinicians have also argued Aimmune was motivated by a financial “bottom line,” which was detrimental to patients who would likely have to pay more for a commercial product.

Moral imperatives and ethical principles have also been invoked by commercial representatives and academic scientists working on IT. They have argued the “right” way to proceed with IT was through the “rigors” of the experimental process (e.g. randomization, blinding, peer review, etc.) to demonstrate safety. An additional example relates to comments by a top-level management official at Aimmune (in an interview prior to the deliberations in 2017), who argued that physicians offering OIT in the clinic were going against the ethical principle of “do no harm” by providing food-based products that contained unstandardized or specified amounts of a food allergen. The claim that private practice physicians were unethically motivated by profits was also articulated by representatives of commercial companies and academic scientists.

In 2017 I was informed by private practice clinicians of IT that those offering the therapy to their patients would meet literally outside of the annual American Academy of Allergy Asthma and Immunology (AAAAI) meeting to discuss their practices and to exchange information about OIT with their colleagues. During the 2017 Canadian Society for Allergy and Clinical Immunology meeting, there was a similar meeting held for those in support of the clinical practice of OIT, during which the spearheaders of the CPG argued that oral immunotherapy was being implemented in other countries as a treatment and that Canadian allergists should establish their own “norms” about the provision of OIT that were potentially different from the practices in the U.S. A desire to normalize oral immunotherapy in the clinic pervaded the discussions at this meeting and provided some of the rhetorical groundwork for the preparation of the CPG in 2019.

Despite the on-going controversies between the aforementioned groups (i.e. academic scientists, commercial representatives, and clinicians), there have been no active attempts to stop the varied practices of OIT in North America and different variations have developed simultaneously. This is rather surprising due to the vociferous claims made for and against both experimental, commercial, and clinical-based IT by different groups during my interviews and observations at clinical and scientific conferences.

It is evident that, despite alignment within the various groups on their different positions regarding whether OIT should be offered as a therapy and how it should be carried out, the claims or arguments had at least until 2019, not been formalized and remained within the purview of “talk” between and among the various practitioners of IT. I did attend pro and con debates about IT and whether it should be offered in the clinic, but these were performative and even for entertainment value at clinical and scientific conferences. The professionals in support of clinical and “real food” oral immunotherapy practice were often situated peripherally to the scientific (experimental and commercial) practices and domains of IT. As a result, and as I will show in subsequent sections, the CPG spearheaders sought to create a cohesive epistemic space for clinical oral immunotherapy that would shift the focus from whether OIT was ready for the clinic or should be taken up, to an emphasis on why and how it should (and could) be implemented in clinical practice.

## The multi-criteria approach (MCA) and the CPG: A “good fit”

As mentioned previously, the creation of an epistemic space for clinical OIT emerged as a result of the “good fit” between the multi-criteria approach practitioners and the needs and desires of the clinical executive group to integrate “other” types of evidence alongside “traditional” evidence in the clinical practice guideline. The notion of the “good fit” is derived from Casper and Clarke’s (1998) exploration of how medical technologies become the “right tools” for the job (i.e. the treatment of various conditions). This approach to studying (medical) technology emphasizes the “work” that is involved beyond the clinic or the lab, to develop or construct a goodness of fit between actors’ desires in a healthcare field or domain. The “good fit” of the MCA approach was not automatic and involved the exploration of other approaches to decision analysis by the clinical leadership group. The following is not an exhaustive outline of the history of the MCA, but highlights the facets of the approach that were emphasized by its practitioners and were shared by the CPG spearheaders.

The multi-criteria approach had previously been used in one other instance of CPG development. Oral immunotherapy served as an additional forum for piloting the framework. The multi-criteria approach for *guidelines* specifically was developed by the methodologists in my research. MCA can be situated within what Hoffman (2018) referred to as the “wider ecology of evaluative research paradigms in biomedicine” (p. 24). There are several evaluative research approaches including for example, Health Technology Assessment (HTA), comparative effectiveness research (CER), and multi-criteria decision analysis (MCDA).^1^ MCDA refers to several analytical techniques (Marsh et al., 2017; Oliveira et al., 2019).

MCA is defined by its explicit critical stance vis-à-vis “traditional” forms of evidence (i.e. randomized controlled trials). The proponents of the MCA (i.e. the methodological support team), the spearheaders of the guidelines, and the participants in the deliberations asserted that “other” forms of evidence should be considered alongside traditional forms of evidence such as randomized controlled trials. Traditional evidence was viewed by both those offering oral immunotherapy and the multi-criteria practitioners as not particularly useful alone nor able to inform healthcare providers or indicate whether they *should* offer a therapy. Thus, the evidence elicited via the multi-criteria approach was thought to remedy a lack of guidance for food allergy professionals. The MCA was furthermore defined as a “new paradigm” and the “second generation” of CPGs to convey the idea that ethical elements and stakeholders’ perspectives differentiated it from other “technical approaches”:

Our approach from the beginning, is a kind of ethical approach, to be in line with the fundamentals of the healthcare system, the public healthcare system, so to take care of the whole population with equity, and everything that comes with that. So, it was not coming from a technical perspective, it was coming from an ethical perspective. We are in opposition with many, many other people in the field of multi-criteria [. . .] who have never looked at it like that. – Executive Group Participant 29

Healthcare technologies, therapies and decisions were framed via the MCA as multi-faceted phenomena (and not a merely technical phenomenon) that implicated several different stakeholders and the framework was conceived as a way to both elicit information (i.e. evidence) from stakeholders and a way to organize “complex” issues. The multi-criteria grid, discussed below, was the cornerstone of the approach and operationalized these principles.

As previously mentioned, the inclusion of ethical dimensions and other stakeholders’ views in CPGs is evidently not actually new. For example, the UK National Institute of Health and Clinical Excellence (NICE) has a long history of involving “other” stakeholders in issues of clinical practice, health technology, and public health (Hoffman, 2019), and in Canada the Ontario Health Technology Assessment Committee (OHTAC) strives to incorporate “ethical” and “social” values in its evidence-based recommendations. Despite this, the guideline developers, by explicitly and formally stating that ethical and stakeholder perspectives were of equal or even greater significance on some criteria than other “traditional” forms of evidence, maintained that their approach to CPG development was unique. The MCA proponents’ views resonate with sociological and other healthcare professionals’ arguments that posit different types of knowledge are needed in guidelines and also lament the absence of methodologies to assess different types of evidence (Wieringa et al., 2018; Zuiderent-Jerak et al., 2012: 2).

Ultimately, the CPG spearheaders united around the notion that the MCA was a “good fit” with OIT due to the other unique or “non-standard” forms of evidence that characterized the field. Participant 30 (a practitioner of the MCA) asserted that the data about OIT was “far beyond” randomized controlled trials and argued it was not a “good idea” to rely on an approach that would solely integrate traditional evidence.

The interactions between the clinical leadership team and the MCA practitioners consolidated a shared sentiment about the inadequacies of “traditional” evidence hierarchies to assess and frame recommendations regarding clinical OIT. The “good fit” between the groups thus represented a challenge to and expansion of the definitions of what constitutes “evidence.” As I will illustrate below, the MCA framework permitted the mobilization of ethical principles and other stakeholder perspectives, some of which have been alluded to in clinicians’ and scientists’ discussions about OIT previously, but had not yet been formally, transparently, and systematically taken up in the form of clinical practice guidance.

### The multi-criteria grid and data collection

A multi-criteria grid was the cornerstone of the performance of the MCA methodology.^2^ The grid was approximately 80 pages in length and was circulated in different iterations to the deliberation committee members, and for referral and guidance throughout the deliberations, as the grid was also situated as a tool to promote “reflection” among participants to expand their perspectives on the items discussed and to enhance *transparency* of the data presentation. The grid was also used to “structure” the data collection process and stakeholders’ interviews.

The grid was distributed in a “working” form at the beginning of the deliberations to all members. The executive team collaboratively completed the grid and presented it at the deliberation meetings in its draft form. The grid distributed during the deliberation meetings was in fact a table that displayed five selected dimensions (socio-political, populational, clinical, organizational, and economic aspects). The first column of the table listed, within each dimension, a number of sub-dimensions. The clinical dimension was the largest section and included sub-dimensions such as clinical efficacy, clinical benefit, adverse events, quality of life, and user empowerment. Within the economic dimension, for example, sub-dimensions included “cost of acquiring and maintaining the intervention for [health and social services].” Next, the adjacent column listed the data gathered through various sources, and a third column listed the “proposed interpretations” of the summaries of all of the data within one facet of each dimension. The “interpretations” were usually 1–3 lines of condensed text. The final publication of the CPG (Bégin et al., 2020) mirrored the presentation of the grid.

Data included in the grid mobilized a variety of “rationales” including evidence from consultations and scientific studies, ethical imperatives, and “other considerations” such as, “standards of clinical care, clinical reasoning, and biological plausibility” (Bégin et al., 2020: 6). Consultations were undertaken with allergists, patients and caregivers, other healthcare professionals and lay “experts.” Data was also collected from the “milieu of care” and economic data was acquired through a “clinical practice offering OIT for [food allergy].” Data collection was complemented by a literature review from multiple sources (e.g. Medline).

### The ethical imperatives

Before elucidating the intricacies of the deliberative process, it is helpful to examine the ethical imperatives that provided the foundation to its development. All the ethical imperatives outlined in the recommendations were not necessarily discussed in detail or debated during the deliberations. In the following sections, I will discuss some of the salient ethical imperatives mentioned during the deliberations. There were a total of 37 recommendations in the CPG that corresponded to the five aforementioned dimensions. The socio-political dimension included 5 recommendations, the clinical dimension 25 recommendations, the organizational dimension 2 recommendations, and the economic dimension 2 recommendations.

Of the 37 recommendations, 9 were “underpinned” by the ethical principle of “non-maleficence” which is defined as the imperative to “not cause or do harm.” Beneficence (relatedly referring to the concept “to do good”) was mentioned three times in the final recommendations and referred to promoting the welfare of patients. A further 9 recommendations were underpinned by the ethical principle of “patient autonomy” or patient agency and 12 recommendations were underpinned by the ethical principle of “equity” (e.g. of “access” to the treatment). “Transparency,” “solidarity” and “sustainability” were invoked once, twice, and five times respectively. Other principles that seemed to re-state these concepts included, “the patient’s best interest,” “patient protection,” “personalized care,” “empowering patients,” the “responsibility of the healthcare system,” and “reducing social inequalities in health.”

## “Normative commitments”

As indicated previously, the focus of the guideline was to establish in a normative sense why and how clinical OIT should be practiced. Thus, in the following section I will elucidate how this was accomplished through a focus on salient dimensions of the deliberations and the final recommendations.

### The shift to how

One of the primary purposes of the CPG was to shift the focus of intra-professional debates from whether OIT should or should not be offered in the clinic, to an emphasis on how it should be taken up and according to which circumstances. The establishment of this premise was particularly important, as commercial company representatives and academic scientists’ claim that it should *not* be practiced in the clinic, peripheralized and de-legitimized the practice of clinical and “real food” oral immunotherapy. The shift to “how” was justified and formalized through several ethical principles and overarching normative frameworks and the initial discussions in the first day of the deliberations focused on re-affirming a consensus among the group that there was a “place” for OIT in the clinic and that it could be useful for patients.

The initial discussions framed the issue of OIT as one of “polarization” constituted by opposing perceptions of “fear” and “hope.” The polarization of the debate about whether IT should or should not be offered was framed as a barrier to its integration in the clinic and as the cause of “confusion” about oral immunotherapy among the general population. Several committee members mentioned the need to promote a change of “culture” in food allergy and it was argued there was a culture of fear surrounding food allergy. It was therefore imperative to catalyze a “different” kind of “attitude” to food allergy, with the primary goals of reducing “fear” and in turn empowering patients and supporting the objectives of the healthcare system. These shared sentiments were articulated in the final published guideline as follows:

It had been proposed as a potential alternative to avoidance throughout the 20th and into the early 21st centuries. . .yet its development had been limited until recently. This is mainly due to the risk of allergic reactions associated with OIT, which is difficult to reconcile with current practice standards that focus on the avoidance of reactions at all cost and are arguably rooted in a culture of fear. (Bégin et al., 2020: 2)

The notion that exposure via OIT was the sole way to remedy “fear” and “confusion” about food allergy among potential patients (and practitioners) was contested during the deliberations by several allergists (both clinician-scientists experimenting with IT and clinicians offering OIT in their clinics). Participants mentioned uncertainties regarding how patients *experienced* avoidance practices and asked questions the potential social or psychological consequences of avoidance, as well as whether better education about food allergy could also be empowering. One allergist wondered whether there were misperceptions about the potential of OIT and therefore about the potential benefits of OIT in the long-term. A patient representative acknowledged that OIT was empowering because it provided knowledge about dealing with and managing adverse reactions to food and took away the fear associated with allergic reactions. The parent, however, was speaking on behalf of her child who was the actual patient; thus, it was impossible to know whether this sentiment was shared by the child. The other patient participant argued that adults had only limited information about OIT.

During discussions about the “how,” it became evident that there were several uncertainties and musings about the impacts of avoidance for food allergic individuals and whether immunotherapy should be framed as the only solution to potential fears regarding food allergy. Despite the uncertainties that emerged during the discussions, avoidance was referred to as a “safe” approach in the final published guideline that “has limited ability to improve a patients’ perception of safety or sense of control of the condition” (Bégin et al., 2020: 2).

As previously mentioned, the framing of the barriers to OIT uptake as constituted solely by a culture of fear and its consequences, did not encapsulate or represent the nuances of experts’ claims and resistance to the implementation of IT in their clinics, as demonstrated by previous interviews during which several academic scientists and commercial representatives argued that IT was not “ready” to be implemented and it should proceed through clinical trials to characterize the risks associated with it and to develop a physiological and immunological understanding of what was occurring. Resistance to the integration of IT in clinical practice by academic scientists undertaking clinical trials was also premised on their own *visions* of what patients wanted, as they asserted patients might prefer a therapy or product that had been FDA-approved and experimentally developed.

The initial discussions during the deliberations were focused primarily on the ethical and normative rationale(s) for opening up an epistemic “space” for OIT. The transformation of the premises of the debate(s) in the domain via the forum at the CPG deliberations was extremely important, as it allowed the participants to circumvent their peripheralization and de-legitimization with a renewed focus on how to operationalize OIT. Despite several uncertainties articulated by participants, the final published guidelines emphasized the fear and polarization of the debates about OIT and the CPG developers’ desire to empower patients vis-à-vis changes in “attitudes” and cultures of fear regarding food allergy.

### Real food and pharmaceutical products

Related to the transformation of the debate into a question of how OIT should be performed, an additional premise was discussed at length about whether the CPG should support the development of food-based and/or pharmaceutical products in OIT. As mentioned previously, this debate has been constituted by moral imperatives espoused by the different participants: academic scientists, commercial representatives, clinician-scientists, and private practice physicians practicing OIT.

As with the shift to how, the participants invoked several moral imperatives explicitly to support the development of clinical and real food oral immunotherapy. The perceived exorbitant costs of the commercial product were framed as antithetical to the *normalization* of food allergen consumption by allergic patients. It was argued that “real food” had the potential to normalize the introduction of food in patients’ diets. Additionally, “real food” was framed as more conducive to the actualization of “personalized care.” Pharmaceutical products were positioned during the deliberations explicitly and implicitly as not necessarily conducive to personalized care. As explicitly stated in the published guideline:

. . . some proponents assert that OIT should only be administered using pharmaceutical products, such as pre-packaged food doses, intended as a life-long treatment, rather than using readily available food for OIT, creating issues that could adversely affect both patients and sustainable development of OIT. . . A fully standardized approach would not be adequate in this context since it cannot respond to each patient’s situation and needs – this calls for research designs on food allergy care to be direct towards answering key questions of personalized care, such as long-term outcomes and real-life needs (Bégin et al., 2020: 7–8).

Within the paradigm of personalized care, real food was positioned as potentially more flexible to meet *patients’* needs and offered the potential to develop “capacity” and expertise of healthcare professionals. Participants argued that the ability to prepare one’s own dosages allowed them to be more flexible and in control of the procedures. Related to this capacity and flexibility, real food was framed as vital to promote patient autonomy:

The goals of OIT can be achieved with many different protocols. There is little evidence that specific dosing schedules are superior to others. Reference protocols can be useful to guide therapy but need to be selected and adapted based on the patient’s specific situation. The final target dose for the therapy should be guided by the patient’s individual clinical response and personal goals, which can range from protection against accidental exposures to small amounts to unrestricted inclusion of the allergen into the diet. [. . .] This recommendation is based on the principles of autonomy to allow patients to achieve their individual goals according to their contexts. (Bégin et al., 2020: 16)

The notion that only clinical care could flexibly incorporate patient desires, was debated during the deliberations. Several allergists noted that clinical trials had “flexibility” built into them, and there was a misperception of patients that they were regimented. This short interaction, despite its brevity, was important because it was one of the only instances during which the facets of experimentalized and pharmaceuticalized immunotherapy were discussed and a more nuanced vision of clinical trials was elaborated. Despite this acknowledgment, the consensus achieved in the deliberations was focused on the inadequacies of the pharmaceutical products.

The discussions during the deliberations did not foreclose the possibility of using pharmaceutical products, but it became clear there was an overwhelming endorsement of non-reliance or non-dependence on pharmaceutical products. This position aligned with previous years’ interviews with clinician-scientists, many of whom were also participants during the deliberations, and of private practice physicians absent from the deliberations who opposed reliance on or use of pharmaceutical products.

Despite the assertion that use of pharmaceutical products were not foreclosed in the final recommendations, there was very little defense of the pharmaceutical product, beyond reference to the ease of consumption and reduced dosage preparation costs. One patient referred to the potential for a food allergy “pill” to reduce the fear associated with food appearance or smell. The disadvantages as indicated above were thought to significantly outweigh the “pros” of the pill referred to by the patient participant in this instance.

Ethical imperatives including references to the autonomy and agency of patients and providers, costs, personalized care and non-reliance on “pharma” pervaded the deliberations and discussions. Despite claims regarding the potential flexibility of experimentalized practices and limited patient input (during the deliberations) concerning the potential advantages of a commercialized product, the aforementioned positionalities about non-reliance or non-dependence on pharma were foregrounded.

### Normalizing allergic reactions in OIT and re-defining food allergy

Whilst the aforementioned dimensions of the OIT CPG were rather explicit regarding ethical and moral imperatives, there was additional focus during the deliberations on re-defining allergic reactions to food as “good” and potentially beneficial for patients.

Individuals who have experienced anaphylactic reactions and/or hospital admissions due to food allergy have frequently been excluded from the practice of oral immunotherapy in clinical trials. Food allergists (both academic scientists and clinicians providing IT) have wondered whether these exclusions are inappropriate because those who have serious reactions could benefit the most from OIT treatment. Concerns about adverse reactions are not trivial, as research has shown individuals with food allergy can experience anxiety and in some cases post-traumatic stress disorder symptoms after anaphylactic reactions (Addolorato et al., 1998; King et al., 2009). Within a paradigm of avoidance, allergic reactions are typically conceived as both undesirable and avoidable, uncomfortable, and potentially dangerous. Patients are told not to ignore their symptoms and seek medical assistance.^3^

A substantial part of the deliberations thus focused on whether food allergies could be defined as “mild” or “severe” and re-framed allergic reactions as a “normal” part of participation in OIT. It was asserted that the term “severity” associated with food allergy should be eliminated, as there was an inability to define “mild” or “severe” reactions a priori. An emphasis on risk factors for experiencing allergic reactions was more desirable. Identification of risks of the likelihood of experiencing a reaction were definable, but not quantifiable on an individual level.

Related to the assertion that food allergies could not be defined by their severity, the deliberation committee sought to re-define *reactions* to food allergens. The experience of reactions was framed as not eliminating the need for avoidance altogether, but was thought to inform and teach patients and professionals how to do avoidance “better.” They were also situated as desirable to demonstrate that OIT was “working” and that patients were “working” on their food allergy.

Clinicians argued that the framing of symptoms as something to be avoided, could have psychological consequences for patients. During the deliberation, there was also an effort to address the meaning of “serious adverse events” (SAE) during OIT, which corresponded to a broader effort to *normalize* adverse reactions. It was argued that while anaphylaxis was defined in some studies as an SAE, it should rather be understood as an effect of engaging in OIT. The use of epinephrine was therefore re-framed as a potentially *necessary* part of OIT and not indicative of a lack of success. Patient representatives were proportionally less involved in the aforementioned discussions, although one argued that OIT helped them develop awareness about how to manage reactions and the other asserted that severity depended on patients’ perceptions and definitions of it.

This element of the deliberation(s) was underpinned by the notion that allergic reactions should be normalized as essential to participation in IT and that it was possible practitioners could (and should) re-frame reactions as potentially beneficial both pedagogically and as “evidence” that patients were “working” on their food allergy. It was clear throughout this particular dimension of the deliberations that it was clinician-directed, with little input from other participants.

## Discussion and conclusion

As mentioned at the outset of this article, both sociologists and medical professionals acknowledge and lament the absence of “other” evidence in the development of clinical practice guidelines. Guideline developers and sociologists have referred to the inadequacies of the “scientization” of guideline development (Moleman et al., 2022). These groups have also noted there is a lack of methodologies available to assess, weigh and integrate “other” types of evidence. Empirical research on how “other” types of evidence are integrated into CPGs has been limited thus far, due to the aforementioned lack of methodologies to operationalize “other” evidence in guidelines.

This case study of the OIT multi-criteria guideline thus represents a departure from the empirical research thus far, as it afforded a unique opportunity to explore how ethical principles and stakeholder perspectives were formally integrated as *evidence* in guideline development. There has been minimal research exploring through which means and contexts, social and ethical principles are integrated into practice guidelines.

This case study re-affirms what has been previously acknowledged in empirical literature on CPGs, namely that guidelines can serve multiple purposes and are not mere technical documents outlining how to perform treatments or interventions in the clinical domain. This case study also takes this proposition a step further to demonstrate how CPGs, like conferences and academic journals for example (Meyer, 2010), can serve as “community-making devices.” The CPG in this case formalized and coalesced what had thus far been scattered claims about the benefits of clinical and real food OIT. The CPG for OIT constructed the rhetorical groundwork to consolidate an “epistemic community” within the domain of food allergy. The CPG for OIT entailed the situated co-production of several normative and ethical premises and commitments to counter de-legitimization and peripheralization from other commercial and academic investigators who have argued IT should not be taken up in a clinical context.

In order to accomplish this, an MCA framework was mobilized by the joint efforts of the spearheaders of the guideline and the methodological support team, that acknowledged “traditional” evidence (e.g. results from randomized controlled trials) were not particularly useful for providing guidance to potential practitioners regarding whether they “should” offer IT to their patients. The operationalization of the MCA framework allowed CPG participants to mobilize ethical principles to highlight shared value-based rationale(s) explicitly and transparently.

As was shown, there were several ethical and moral imperatives mentioned in the final guideline, but a strong emphasis was placed on the notions of patient autonomy, empowerment, and “personalized care.” Emphasis was also placed on issues of affordability and costs to the healthcare systems vis-à-vis the use of non-commercialized products or non-reliance on commercialized products. Providers’ needs and desires were framed largely in alignment with patient needs, such as the desires for autonomy of practice, with the aims of developing “capacity” among potential OIT providers. The mutually inclusive representation of patient and provider desires harkens back to Smith’s (2015) observation that food allergists have historically cultivated trusting and close relationships with patients and have been dependent on their patients’ observations and records to perform diagnostics and treatments.

This case study is unique among empirical investigations thus far due to its explicit and “transparent” presentation of the ethical principles underpinning its development. Sociologists investigating ethical principles via CPG and other scientific and biomedical technologies have thus far had to “uncover” or “reveal” what are typically “latent” normative and ethical ideals or tropes. Ethical principles in this particular case in contrast were considered and presented transparently alongside other forms of evidence.

This case study also elaborates on the wealth of research that posits laboratories are only “partial” sites of knowledge production and development and which has begun to explore the “devices” that make epistemic communities and collectives (e.g. conferences, etc.). This case study posits that this particular CPG was a community-making device that emphasized the “values” of ethical evidence and both explicated and consolidated these value(s) in support of the uptake of clinical and real food OIT.

While the MCA approach may be encouraging to “critical” sociologists due to its inclusion of “other” forms of knowledge and some healthcare professionals alike because of its explicit integration of ethical imperatives, there is also some uncertainty about how this type of guideline or tool will be taken up among other “hierarchies of credibility” (Clarke and Fujimura, 1992). For example, it is uncertain how it will be taken up and interpreted by those who have been staunchly opposed to the development of IT in its non-experimental or commercial forms, by “other” stakeholders including patients on a larger scale and by those who may be responsible for determining whether to fund IT. Indeed, the *same* ethical and moral imperatives have been taken up and invoked by commercial representatives who have asserted those offering OIT to their patients are doing so without regard for patient safety, but for the purposes of a financial “bottom line.” Academic scientists undertaking multi-site clinical trials for IT have also argued that the “right” and “moral” way of engaging in IT is through the rigors of clinical trials. Additionally, these groups have also attributed the development of the different modalities of IT to the “needs” of potential patients and thus have different visions for what patients might desire. The OIT CPG will thus have to contend with the apparent flexibility of ethical and moral principles and “visions” of what patients desire as espoused by academic scientists and commercial representatives.

Furthermore, as has been noted by several sociologists studying CPGs, the methodologies for evidence synthesis from the various “rationales” outlined throughout this article were not explored or explained in detail during my research. During the deliberations, some views on various topics or questions were inevitably siphoned off or out of the final published guideline, despite the uncertainties articulated around many topics. It is thus still desirable to investigate how and through which means “interpretations” and evidence synthesis occurs particularly when several kinds or types of evidence are formally integrated into CPG development and recommendations. The need to understand how evidence is synthesized becomes particularly important for example in the context of aspects in which patients are not involved and/or have no prior experience on which to base their feedback and insight. For example, the economic dimensions of the discussions were elements of the CPG process that patients could not meaningfully contribute to. As noted, the discussion regarding the normalization of allergic reactions was also heavily clinician-dominated and thus it is unknown how patients themselves will perceive these recommendations and how their feedback or insights informed this discussion.

The notion that all “stakeholders” of CPGs come to the “table” with “vested interests,” is something that was not explicitly discussed in detail in the OIT CPG development process. There were efforts made by the CPG spearheaders to exclude those who were too “pro” IT (i.e. those who derived a significant amount of income from performing private practice OIT) and/or those who were too “con” clinical OIT (i.e. the academic scientists who argue IT should not be offered in the context of clinical care and the commercial representatives).

Additionally, in line with the suggestions of Moleman et al. (2022), it is helpful and perhaps necessary going forward in the development of CPGs, to emphasize and explicate the applicability of clinical practice guidelines to their domains of care and practice. Whilst the formal and laudable integration of both patient and ethical principles vis-à-vis the MCA approach responds to the calls by sociologists and medical professionals, the uptake and legitimacy of this type of guideline could acquire further usefulness and credibility through explicit discussions of and visions for how it can and will inform actual clinical practice.

## Notes

1. My informants suggested that I refer to the framework that structured the CPG process as a multi-criteria approach (MCA) which was differentiated from MCDA. The change of terminology refers to an effort by the methodologists to re-define MCDA as a process that did not necessarily entail a “decision” as per the use of MCDA to make policy or healthcare decisions about funding, but was rather a methodology used to structure and guide the CPG development process.

2. The methodology discussed here describes the CPG and not the methodologies I used to collected data.

3. See: https://foodallergycanada.ca/food-allergy-basics/preventing-and-treating-allergic-reactions/reaction-signs-and-symptoms/
